# Using genetic association data to guide drug discovery and development: review of methods and applications

**DOI:** 10.1016/j.ajhg.2022.12.017

**Published:** 2023-02-02

**Authors:** Stephen Burgess, Amy M Mason, Andrew J Grant, Eric AW Slob, Apostolos Gkatzionis, Verena Zuber, Ashish Patel, Haodong Tian, Cunhao Liu, William G Haynes, G Kees Hovingh, Lotte Bjerre Knudsen, John C Whittaker, Dipender Gill

**Affiliations:** 1 MRC Biostatistics Unit, School of Clinical Medicine, University of Cambridge, Cambridge, UK; 2 Cardiovascular Epidemiology Unit, Department of Public Health and Primary Care, University of Cambridge, Cambridge, UK; 3 MRC Integrative Epidemiology Unit, University of Bristol, Bristol, UK; 4 Department of Epidemiology and Biostatistics, School of Public Health, Imperial College London, UK; 5 MRC Centre for Environment and Health, School of Public Health, Imperial College London, UK; 6 UK Dementia Research Institute at Imperial College, Imperial College London, UK; 7 Novo Nordisk Research Centre Oxford, Novo Nordisk, Oxford, UK; 8 Radcliffe Department of Medicine, University of Oxford, Oxford, UK; 9 Department of Vascular Medicine, Academic Medical Center, Amsterdam University Medical Centers, University of Amsterdam, Amsterdam, The Netherlands; 10 Global Chief Medical Office, Novo Nordisk, Copenhagen, Denmark; 11 Chief Scientific Advisor Office, Research and Early Development, Novo Nordisk, Copenhagen, Denmark

## Abstract

Evidence on the validity of drug targets from randomized trials is reliable, but typically expensive and slow to obtain. In contrast, evidence from conventional observational epidemiological studies is less reliable, due to the potential for bias from confounding and reverse causation. Mendelian randomization is a quasi-experimental approach analogous to a randomized trial that exploits naturally-occurring randomization in the transmission of genetic variants. In Mendelian randomization, genetic variants that can be regarded as proxies for an intervention on the proposed drug target are leveraged as instrumental variables to investigate potential effects on biomarkers and disease outcomes in large-scale observational datasets. This approach can be implemented rapidly for a range of drug targets to provide evidence on their effects and thus inform on their priority for further investigation. In this review, we present statistical methods and their applications to showcase the diverse opportunities for applying Mendelian randomization in guiding clinical development efforts, thus enabling interventions to target the right mechanism in the right population group at the right time. These methods can inform investigators on the mechanisms underlying drug effects, their related biomarkers, implications for the timing of interventions, and the population subgroups that stand to gain most benefit. Most methods can be implemented using publicly available data on summarized genetic associations with traits and diseases, meaning that the only major limitations to their usage are the availability of appropriately powered studies for the exposure and outcome, and the existence of a suitable genetic proxy for the proposed intervention.

## Introduction

**Figure 1:**
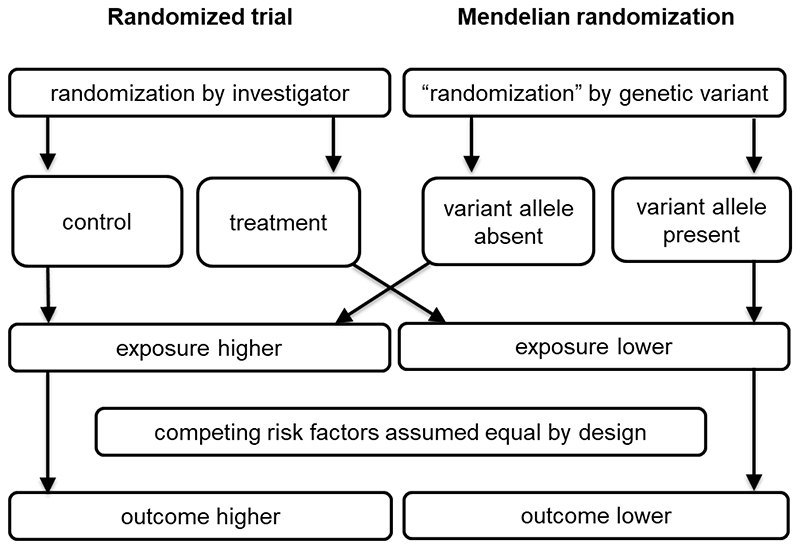
Schematic diagram illustrating analogy between Mendelian randomization and randomized trial: in a randomized trial, the population is split into control and treatment groups at random by the investigator; in Mendelian randomization, the population is divided into groups based on a genetic variant (a “natural experiment”).

**Figure 2:**
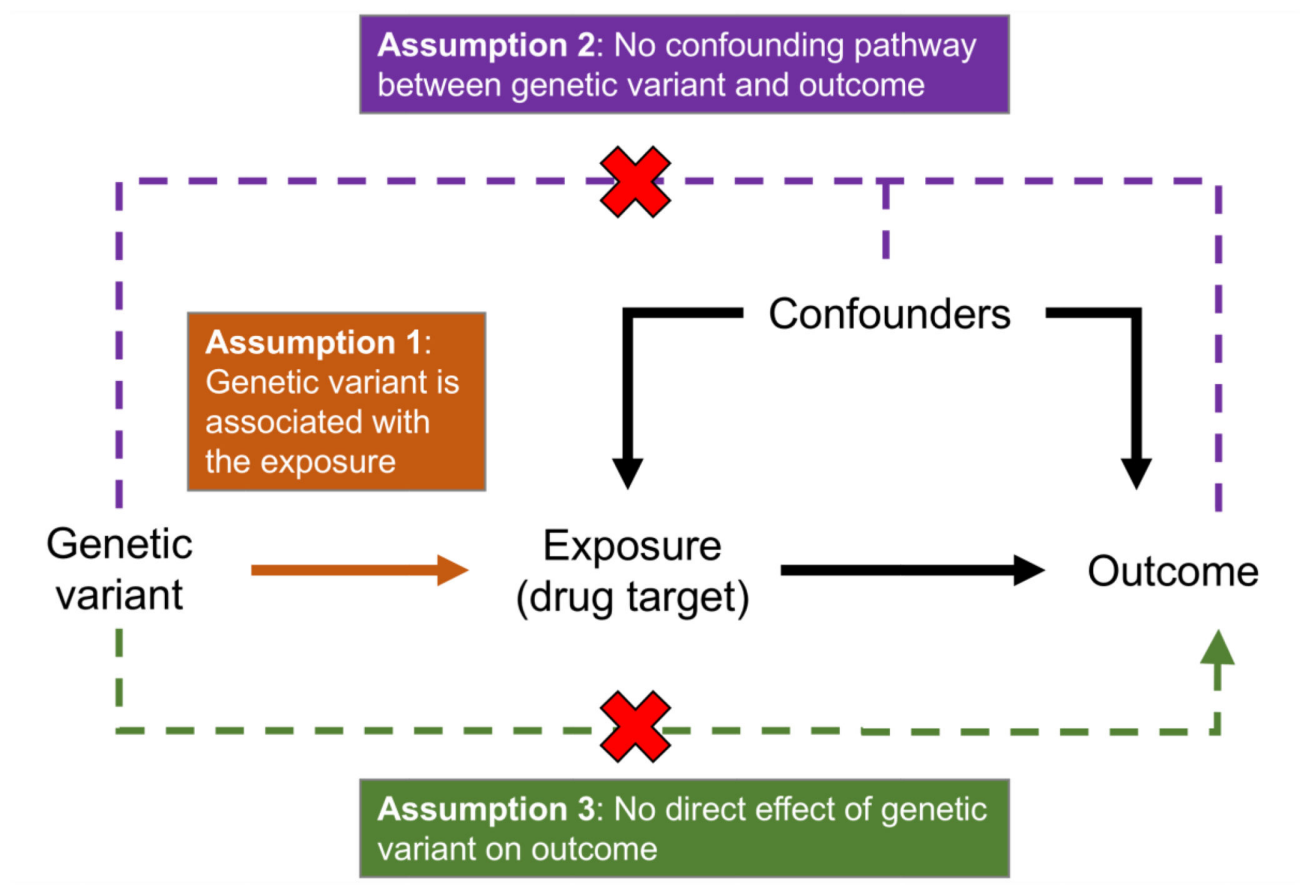
Schematic diagram illustrating the instrumental variable assumptions. For a genetic variant to be a valid instrumental variable, it must: 1) be associated with the exposure (that is, population subgroups defined by the genetic variant have different average levels of the exposure); 2) not be associated with the outcome by a confounding pathway (that is, population subgroups defined by the genetic variant have similar average levels of competing risk factors); and 3) not have a direct effect on the outcome (that is, any influence of the genetic variant on the outcome operates via the exposure). If the genetic variant satisfies these assumptions, then any association between the variant and the outcome must be due to a causal effect of the exposure.

The availability of data on genetic associations from large-scale epidemiological studies offers the opportunity to improve the efficacy and efficiency of drug development. Genetic variants that affect the function of a gene may be used to provide insight into the efficacy, adverse effects, and repurposing potential of interventions that perturb the corresponding protein in specific population groups (1). Mendel’s second law (“the independent segregation of alleles at conception”) means that these genetic variants should not associate systematically with respect to confounding variables, creating a natural experiment analogous to a randomized trial (2) (Figure 1). The use of genetic variants as instrumental variables (Figure 2) to assess causal relationships from observational data is known as Mendelian randomization (3, 4).

The relevance of Mendelian randomization to drug discovery efforts has previously been discussed at length (5–7). The majority of drug targets are proteins, which are coded for by genes. Genetic variants can therefore be used as proxies for studying the effect of pharmacologically perturbing these protein drug targets. Due to the random allocation of genetic variants at conception, variants are typically distributed independently of potential confounders conditional on the parental genotype. This independence has been shown empirically to hold marginally at a population level for many variants (8, 9). Therefore, aside from associations arising due to population stratification or linkage disequilibrium, genetic variants should only be associated with traits that they affect, and so should be independent of confounding factors. Furthermore, the fixed nature of the genotype means it cannot be influenced by environmental variables (10), thus reducing the possibility of spurious results due to confounding by such factors, and ensuring genetic associations are protected from bias due to reverse causation (11). Therefore, compared with conventional observational epidemiological analyses, Mendelian randomization analyses have the potential to provide more reliable insights into causal relationships (12). The human relevance of such analyses can offer considerable advantages over animal models, from which findings may not be directly translatable (13).

Systematic reviews indicate that drug targets with human genetic evidence are more than twice as likely to progress into clinical practice (14, 15). Furthermore, the availability of large-scale genetic association data and genotyped biobanks offers the potential for time- and cost-efficient investigations (16). Indeed, two-thirds of drugs approved in the US in 2021 have evidential support from human genetics (17). These advantages have led to the widespread adoption of genetic data as a key evidence source in drug discovery, and generation and analyses of genetic data have become a key strategic element for many pharmaceutical companies.

In this review, we present ways in which methodological developments in Mendelian randomization and other related genetic analyses can be used not only to select between promising targets, but also to inform all stages of drug development (18). The ultimate judge of therapeutic effectiveness is a clinical trial. However, while a necessary part of drug development, trials can be expensive and time-consuming (19). Many Mendelian randomization analyses can be performed rapidly and efficiently using existing data resources. Genetic analyses that address questions of translational relevance can guide trial design by gathering focused evidence on what are the best targets for intervention, when is the optimal time to intervene, and who would most benefit from intervention. This enables trials to be prioritized that have the greatest chance of an informative outcome.

We focus on nine areas where genetic evidence can impact drug development: 1) target validation; 2) clarification of causal pathways; 3) identification of relevant outcomes; 4) elucidation of complex mechanisms; 5) uncovering of tissue specificity; 6) investigation of heterogeneity in response to treatment; 7) ancestry-specific considerations; 8) prediction of the magnitude of effect of an intervention; and 9) understanding the impact of timings of interventions. In each case, we consider methodological developments and provide practical examples, focusing on how these methods have been used in the existing literature. A summary of some relevant examples is given as Table 1.

## 1 Target validation

We initially discuss conventional Mendelian randomization analyses, and proceed to consider related and complementary approaches: use of colocalization, genetic predictors of drug response, and rare variants.

Two categories of Mendelian randomization investigations are those using variants from a single genetic region and those using variants from multiple genetic regions (polygenic analyses) (20). Mendelian randomization analyses for target selection and drug development typically use genetic variants from a single genetic region, the region around the gene that encodes the protein target for investigation. Such analyses have been called “*cis*-Mendelian randomization” analyses, as genetic variants in a relevant coding gene region are known as *cis*-variants (6). When investigating drug targets, the exposure is taken as a measure of pharmacological perturbation of the relevant drug target. Choice of genetic region is crucial to the validity of the investigation; if variants in the genetic region do not replicate the effect of the drug, then the investigation has limited value (5).

As an example, a *cis*-Mendelian randomization investigation considered coagulation factor X as a risk factor for various cardiovascular diseases using a genetic variant in the *F10* gene region that had previously been shown to associate with plasma levels of activated factor X (FXa) (21). Given the use of FXa inhibitors for preventing deep venous thrombosis and pulmonary embolism (22), genetic associations with these outcomes provided confirmatory evidence of the efficacy of interventions on this pathway. A non-significant association with coronary artery disease (despite greater number of cases) suggests that prevention of this outcome is lower priority for pursuit through FXa inhibition as a monotherapy.

Although several of the examples included in this review focus on cardiovascular diseases, Mendelian randomization investigations have been performed for many other outcomes. The bias towards cardiovascular disease in this review (which reflects a bias in the literature (23)) is primarily because: 1) there are several modifiable risk factors for cardiovascular disease that are suitable for consideration as exposures in Mendelian randomization; and 2) summarized data from large consortia were released earlier for cardiovascular diseases than for most other diseases.

If there are multiple candidate variants in a genetic region, investigators may consider including multiple variants in their analysis. Variants chosen for inclusion in a *cis*-Mendelian randomization analysis should be conditionally independent predictors of the putative causal trait, which we refer to as the exposure. That is, each variant included in the analysis should be associated with the exposure in a model adjusting for other included variants. If a Mendelian randomization analysis is performed using summarized data, any correlation between variants should be accounted for in the analysis (24). If two variants are in perfect linkage disequilibrium, estimates will be no more precise when using both variants compared with using one. Variants in partial linkage disequilibrium can increase the power of an analysis if they explain independent variation in the exposure (25).

Selecting too many correlated variants can lead to numerical instability in Mendelian randomization estimates, as multicollinearity between variants makes estimates highly sensitive to misspecification of the variant correlation matrix (26). One option to mitigate this issue is to employ a variable selection method from the fine-mapping literature to select a small number of variants with independent signals, such as Bayesian stochastic search (4). An alternative approach is to perform dimension reduction on the matrix of genetic variants, and use a small number of principal components or factors from this matrix as instruments (26, 27). However, unless additional variants explain a substantial fraction of additional variance in the exposure, gains in power over analyses based on the lead variant alone are likely to be small (28).

A particular concern to the validity of Mendelian randomization analyses is pleiotropy (sometimes called “horizontal pleiotropy”), defined here as the association of a genetic variant with a trait which is not on the causal pathway from the variant to the outcome through the exposure (29). While there is a wide variety of Mendelian randomization methods that are robust to some degree of pleiotropy (30), these methods typically rely on the availability of variants in multiple gene regions, hence allowing the consistency of findings across these variants to be investigated for a polygenic Mendelian randomization analysis. As an example, several genes are implicated in the synthesis and metabolism of vitamin D. Variants in four distinct gene regions linked to vitamin D were shown to associate concordantly with the risk of multiple sclerosis, providing evidence of a potential protective effect of vitamin D on multiple sclerosis risk (31). However, these methods typically are of limited benefit when variants are all located in a single genetic region, because the assumption of instrument validity is not independent for different variants in such a single region.

An alternative approach that can be used to assess the robustness of a *cis*-Mendelian randomization finding is colocalization. Colocalization is a method developed in the context of genome-wide association studies (GWAS) to distinguish between two scenarios at a genetic region containing variants associated with two traits: 1) the traits are affected by the same variant(s), or 2) the traits are affected by different variants (32). If colocalization is applied to the exposure and outcome from a Mendelian randomization analysis, the latter scenario is likely to represent violation of the Mendelian randomization assumptions (Figure 2), as a genetic predictor of the exposure could be associated with the outcome via linkage disequilibrium with a distinct variant that influences the outcome. For example, colocalization analyses suggest that the causal variants for LDL-cholesterol and Alzheimer’s disease at the *APOE* locus are distinct, indicating that different mechanisms underlie these associations (33). A related example is at the *GLP1R* locus, where the lead signal for bodyweight reduction is not the same as that for lowering elevated blood glucose (33), suggesting distinct signalling mechanisms underlie the effect of glucagon-like peptide 1 receptor (GLP1R) agonism on these two outcomes (34).

A limitation of the use of colocalization as a sensitivity analysis for Mendelian randomization is that colocalization methods (in particular, the *coloc* method (35) and its derivatives) require the presence of a genetic variant that is strongly associated with each trait in the given genetic region to conclude that there is colocalization. If a genetic variant is associated with the outcome at a relatively weak level of statistical significance (say *p* = 0.005), then the *coloc* method under its default prior settings will typically conclude that there is no causal variant for the outcome (in the language of *coloc*, this is the hypothesis *H_1_*), rather than evidence for the hypothesis of distinct causal variants (*H_3_*) or a shared causal variant (*H_4_*; this is colocalization) (33). Such a finding does not provide strong evidence either in favour of or against the validity of the Mendelian randomization assumptions.

As an example, two genetic variants in the *F11* gene region that are associated with circulating levels of coagulation factor XI have been shown to be associated with risk of ischaemic stroke, and in particular the cardioembolic subtype (36). In a separate investigation (37), variants in the *F11* gene region were shown to colocalize for circulating levels of factor XI and venous thromboembolism using the *coloc-SuSiE* method (38), an extension of the standard *coloc* method that allows colocalization to be detected when there is more than one causal variant at a given locus. This supports the findings of Phase II trials, which have shown evidence for the beneficial effect of factor XI inhibition on venous thromboembolism risk (39).

As a complementary approach to strengthen target validation, investigators considered the impact of statins on cardiovascular diseases, not by using genetic variants in the *HMGCR* gene that encodes the target of statin therapy, but instead by using a score comprising genetic variants that predict statin efficacy (see Section 6) (40). Amongst statin users, this score was inversely associated with risk of myocardial infarction and peripheral vascular disease, and positively associated with intracerebral haemorrhage, mirroring results from statin trials (41, 42) and genetic analyses using variants in the *HMGCR* gene (43). This is an example of triangulation: the use of distinct approaches to address the same question (44). Amongst statin non-users, the score was not associated with any of these diseases. Statin non-users represent a natural negative control group, as we would not expect genetic variants that influence the efficacy of statins to affect disease outcomes amongst individuals who do not use statins. A limitation of this analysis is that the restriction to statin users could induce selection bias. However, the impact of mild selection effects on Mendelian randomization estimates is typically not substantial (45).

Mendelian randomization investigations can be performed using rare genetic variants, although specific methodological approaches are recommended when considering evidence from multiple rare variants. To assess associations with rare variants, it is common to consider collapsing analyses that combine information on several variants into a single test (46). If genetic associations are considered for individual variants in isolation, there may not be enough power to detect an association. By combining information across variants in a gene region, power can be increased. Several approaches have been considered, including burden tests, which combine multiple variants into a single burden variable and assess associations of the burden variable, and kernel-based test methods, which combine test statistics for each variant using a kernel matrix. The sequence kernel association optimal unified test (SKAT-O) method combines these two approaches in a data-driven way (47). Rare variant approaches have been used in a hypothesis-free way to search for associations across the genome (48), and in a focused way, for example to demonstrate associations of variants in the *CIDEB* gene with liver disease (49). The latter investigation used the REGENIE method (50), which has been developed to perform such analyses in large datasets. A detailed review of rare variant methods is beyond the scope of this paper; a recent review can be found here (51).

A critical question when performing a Mendelian randomization analysis is how to choose which genetic variants to include in the analysis: first, which gene regions to focus on, and second, which variants from these regions to include in the analysis. Mendelian randomization analyses are most reliable when variants are selected on the basis of their biological relevance to the exposure, or more specifically, their relevance to the proposed intervention on the exposure (20). For this reason, drug target Mendelian randomization analyses typically focus on a single gene region. Selection of variants from a gene region may be guided by functional insight, or associations with levels of a circulating biomarker, a relevant protein, or gene expression in a relevant tissue or cell type. The optimal approach in any case will depend on the specific investigation and the available data. Discussion of these considerations in the context of relevant examples can be found in (5) and (52).

## 2 Clarifying causal pathways

**Figure 3:**
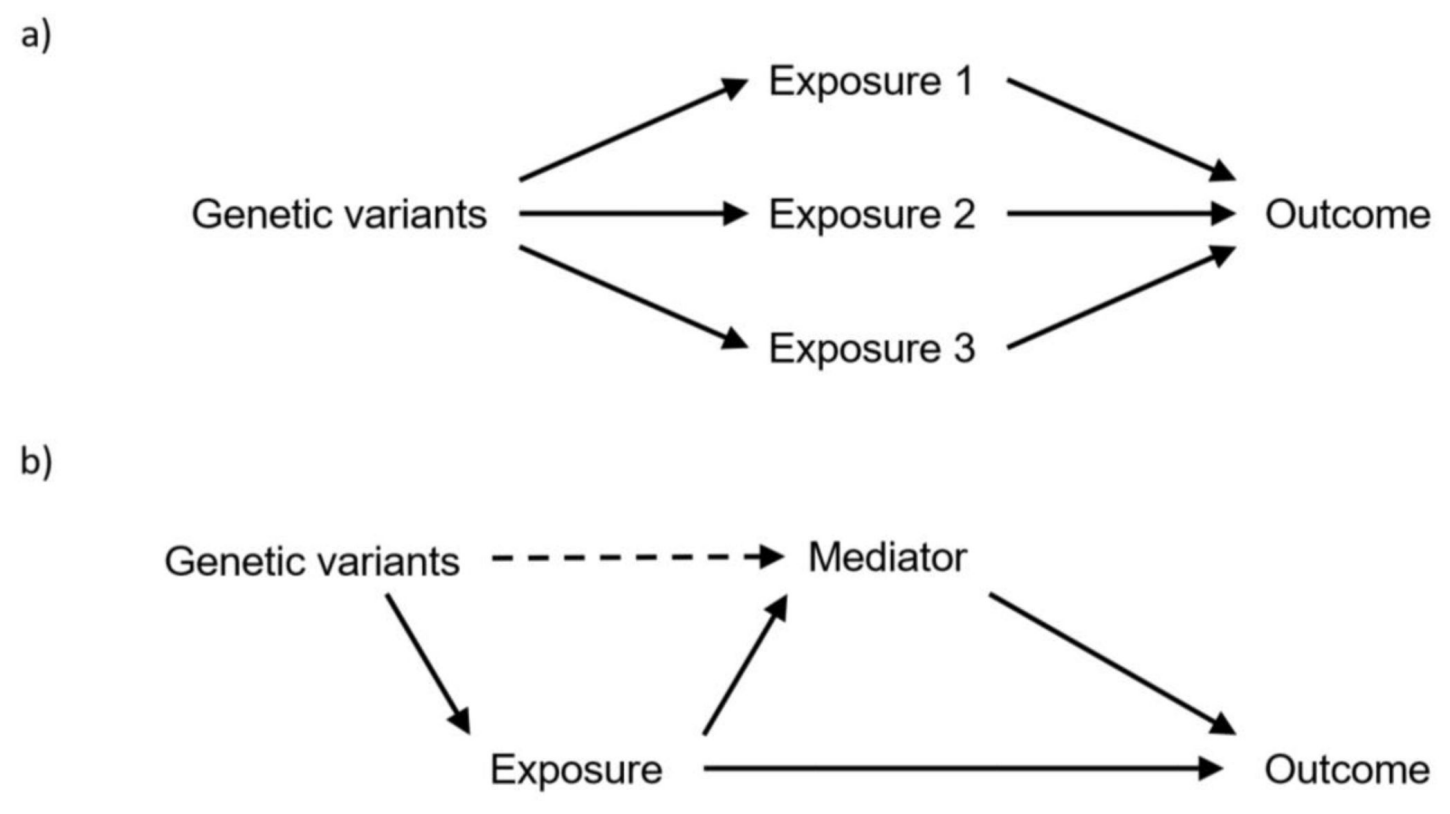
Schematic diagram illustrating two scenarios in which multivariable Mendelian randomization can be used: a) to disentangle the effects of related traits with shared genetic predictors, and b) to assess mediation in the effect of an exposure via a proposed mediator.

Although Mendelian randomization provides evidence about the causal nature of an exposure for a disease outcome, more detailed analyses are required to understand the causal pathway by which the exposure may influence the outcome. A relevant approach here is multivariable Mendelian randomization, the use of genetic variants that are associated with multiple exposures to estimate the effect of each exposure on the outcome (53, 54). While standard (that is, univariable) Mendelian randomization assesses whether genetically-predicted levels of an exposure are associated with the outcome in a univariable model, multivariable Mendelian randomization assesses whether genetically-predicted levels of multiple exposures are associated with the outcome in a multivariable model (55). Two scenarios in which this method can be used are: 1) when there are several related traits with shared genetic predictors, and 2) to assess mediation of the effect of a complex trait via one or more proposed mediators (Figure 3) (56).

As an example of the first scenario (related traits), it is difficult to find genetic predictors of high-density lipoprotein (HDL) cholesterol concentrations that are not also associated with low-density lipoprotein (LDL) cholesterol and/or triglyceride concentrations. However, while polygenic univariable Mendelian randomization analyses suggest that genetically-predicted levels of HDL-cholesterol are associated with coronary artery disease risk, this association attenuates substantially in multivariable Mendelian randomization analyses. The univariable analysis is subject to potential bias, as several of the genetic predictors of HDL-cholesterol concentrations have pleiotropic effects on the outcome via LDL-cholesterol and/or triglycerides. Conditional on genetically-predicted LDL-cholesterol and triglyceride concentrations, the association between genetically-predicted HDL-cholesterol concentrations and coronary artery disease risk is attenuated toward the null (57). This suggests that HDL-cholesterol by itself is not a worthwhile target for pharmacological intervention in a general population.

An extension of this method is the Mendelian randomization Bayesian Model Averaging (MR-BMA) method, which extends multivariable Mendelian randomization to consider large numbers of traits, comparing the evidence for different sets of exposures as causal risk factors (58). An analysis considering genetic predictors of 30 lipidomic measurements concluded that the most plausible causal model for those data had apolipoprotein B as the sole risk factor affecting coronary artery disease. This suggests that differences in coronary artery disease risk are proportional to the change in the number of hepatically-derived cholesterol carrying lipoprotein particles, not the concentration of LDL-cholesterol (59). A similar conclusion was reached investigating lipid predictors of cardiovascular risk reduction in response to pharmacological agents in clinical trials (60). Genetic analyses have yielded similar conclusions for peripheral artery disease (61). These findings have implications for the choice of targets for lipid-lowering therapies and the measurement of lipid traits in clinical trials for cardiovascular disease.

As an example of the second scenario (mediation), previous Mendelian randomization investigations have demonstrated evidence for body mass index (BMI) as a causal risk factor for coronary heart disease (62). However, the mechanism linking BMI to coronary heart disease risk is unclear. Multivariable Mendelian randomization analyses have suggested that the effect of BMI on coronary heart disease risk is partially mediated via systolic blood pressure and type 2 diabetes propensity (63). This implies that much of the cardiovascular benefit of BMI lowering could be achieved by reductions in blood pressure and risk of type 2 diabetes. Such insight also has direct relevance for informing of the population that stands to gain greatest cardiovascular risk benefit from bodyweight reduction.

Multivariable Mendelian randomization is difficult to implement for *cis*-Mendelian randomization analyses, as it is necessary to have at least as many independent genetic variants as there are traits in order to estimate the effect of each trait (64). Additionally, it is necessary to have variants that differ in their relative strength of association with the traits; if genetic associations with two traits are perfectly proportional, then it is not possible to distinguish between the traits in a multivariable model (55).

Multivariable Mendelian randomization could have utility for *cis*-Mendelian randomization if multiple traits are associated with variants in a particular genetic region linked with a disease outcome. A potential scenario where this may occur is for protein traits at a “gene cluster”, a region of the chromosome containing several adjacent genes, that contains one or more GWAS hits, such as the interleukin-1 receptor cluster that contains variants associated with various autoimmune diseases (65, 66). A recent methodological development extended dimension reduction methods for variant selection to the multivariable *cis*-Mendelian randomization setting (67). While in univariable *cis*-Mendelian randomization, including multiple variants may increase power slightly; in multivariable *cis*-Mendelian randomization, including multiple variants is necessary to disentangle the various putative causal traits. This method was used to show that, despite the presence of multiple protein associations at the locus, the most likely causal risk factor for cardioembolic stroke at the chemokine receptor gene cluster is monocyte chemoattractant protein-1 (67).

## 3 Identifying relevant outcomes

An efficient way of addressing unmet medical need is the repurposing of existing drugs for novel disease outcomes. If genetic variants can be found that proxy a particular pharmacological intervention and are valid instrumental variables, then genetic evidence for the effect of the intervention on a range of outcomes can be assessed in a phenome-wide association study (68, 69). Outcomes can include continuous traits and diseases: intermediate biomarkers indicating efficacy of the treatment (which could therefore be used in early-phase trials as proxies for a disease outcome), positive and negative controls, putative additional indicated disease outcomes, and safety signals.

For example, genetic variants in the *IL6R* gene region that can be considered as proxies for intervention on interleukin-6 signalling are associated with levels of downstream inflammatory biomarkers (C-reactive protein, fibrinogen) similarly to tocilizumab, an interleukin-6 receptor inhibitor (70). Tocilizumab is used in the treatment of rheumatoid arthritis, which is therefore a positive control outcome. These *IL6R* variants are associated with rheumatoid arthritis, and with coronary artery disease in the same direction, suggesting that interleukin-6 receptor inhibition may also reduce risk of cardiovascular disease (71). These same variants have also been shown to be associated with reduced risk of COVID-19 and COVID-19 hospitalization (72, 73); tocilizumab was subsequently shown to be an effective treatment for reducing COVID-19 severity in the RECOVERY trial (74).

Wide-angled Mendelian randomization analyses (that is, analyses considering a broad set of outcomes) could help define the therapeutic effects of an intervention, and hence the primary outcome of a clinical trial. Phase III trials are currently underway for antisense oligonucleotides that substantially reduce lipoprotein(a) concentrations (75). Genetic variants in the *LPA* gene region are strongly associated with coronary heart disease, displaying a dose-dependent relationship on the log-linear scale (76) (see Section 8). Variants in this gene region are also associated with risk of ischaemic stroke, peripheral artery disease, abdominal aortic aneurysm, and aortic stenosis, but not with haemorrhagic stroke (77), suggesting that haemorrhagic stroke should not be considered as part of the primary efficacy endpoint for lipoprotein(a) trials.

An example of a potential safety signal is the genetic association of variants in the *CETP* gene region with age-related macular degeneration (AMD) risk (78, 79). Cholesteryl ester transfer protein (CETP) inhibitors are being developed for treatment of cardiovascular diseases (80), and vigilance for increased risk of AMD incidence or progression in treated patients may be appropriate.

## 4 Elucidating complex mechanisms

Genetic associations can be used to untangle complex biological mechanisms. As a simple example, genetic predictors of C-reactive protein concentrations in the *IL6R* gene region are associated with coronary artery disease risk (70), but those in the *CRP* gene region are not (81). The *IL6R* gene region encodes interleukin-6 receptor; interleukin-6 is upstream of C-reactive protein in the inflammatory cascade. The genetic associations suggest that targeting interleukin-6 pathways may reduce coronary artery disease risk, but targeting C-reactive protein directly will not.

**Figure 4:**
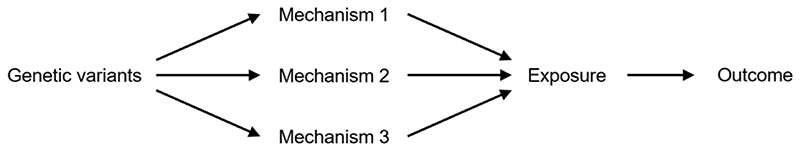
Schematic diagram illustrating a potential explanation leading to clustering of genetic variants associated with an exposure. Two approaches have been proposed to cluster genetic variants in a Mendelian randomization analysis: 1) clustering based on variant-specific Mendelian randomization estimates; and 2) clustering based on associations with related traits.

A typical feature of polygenic Mendelian randomization analyses is heterogeneity amongst genetic predictors of an exposure in their associations with the outcome. While this can be a sign of specific variants having pleiotropic associations (29), it could instead reflect the presence of multiple causal mechanisms by which the exposure influences the outcome, particularly if the Mendelian randomization estimates for different variants cluster around distinct values. Such clusters may reflect distinct components of the exposure that have different effects on the outcome, different mechanisms of intervention on the exposure, or distinct causal pathways passing via the exposure (82) (Figure 4). Untangling these mechanisms could help pinpoint specific aspects of a complex exposure that could be intervened on to reduce disease risk (83).

Two methods with relevance to Mendelian randomization have been proposed for the clustering of genetic variants associated with a particular exposure: one that clusters variants based on their associations with the outcome, and another that clusters variants based on their associations with multiple traits, but typically does not include the outcome among these traits.

The first method, *MRClust,* finds groups of variants with similar Mendelian randomization estimates for the effect of the exposure on the outcome, but then requires the user to explore genetic associations with different traits to interpret the clusters (82). For example, although most genetic predictors of increased BMI were positively associated with type 2 diabetes risk, a minority cluster of genetic variants was associated with increased BMI but decreased type 2 diabetes risk (84). A shared feature of this cluster was associations with increased birthweight, suggesting a dichotomy in effects on type 2 diabetes risk between genetic variants that predispose an individual to excess adiposity in later life versus those that predispose an individual to large size from birth (85).

The second method, *NAvMix,* finds groups of variants having similar proportional associations with a range of traits (86). If the outcome is not included in the clustering algorithm, then we can test whether the Mendelian randomization estimates for the outcome based on different clusters differ. For example, genetic predictors of BMI were divided into five clusters based on their associations with nine cardiovascular traits. While three of these clusters had positive Mendelian randomization estimates for the outcome of coronary heart disease, the fourth cluster had a null estimate, and the fifth cluster had a negative estimate. The fifth cluster, which was also the smallest cluster, was associated with cardiovascular traits in a pattern that has previously been described as “metabolically favourable adiposity”, namely increased HDL-cholesterol concentrations, and decreased systolic blood pressure, triglyceride concentrations, waist-hip ratio and type 2 diabetes propensity (87). This cluster also differed from others in its associations with levels of inflammatory biomarkers (86). This implies there may be targets that increase bodyweight but decrease other biomarkers of cardiometabolic disease, and have favourable overall effects on cardiovascular disease risk.

An alternative approach to unravel complexity is to perform dimension reduction on a set of related traits. This can be used to summarize high-dimensional data on a complex phenotype into meaningful composite variables. For example, it is unlikely that a single component of body composition is causal for metabolic disease, but rather the agglomerative effect of a mechanism such as adiposity, which influences many anthropometric traits. Principal component analysis methods have been applied to reduce multiple body composition measures into four main components, representing body size, adiposity, predisposition to abdominal fat deposition, and lean mass, which were then investigated in Mendelian randomization analyses (88). Another context where this approach could be used is to partition imaging data into components, using methods such as sparse principal component analysis, which creates components that are sparse (that is, they are only calculated based on a subset of all available variables), and hence are more interpretable (89).

**Figure 5:**
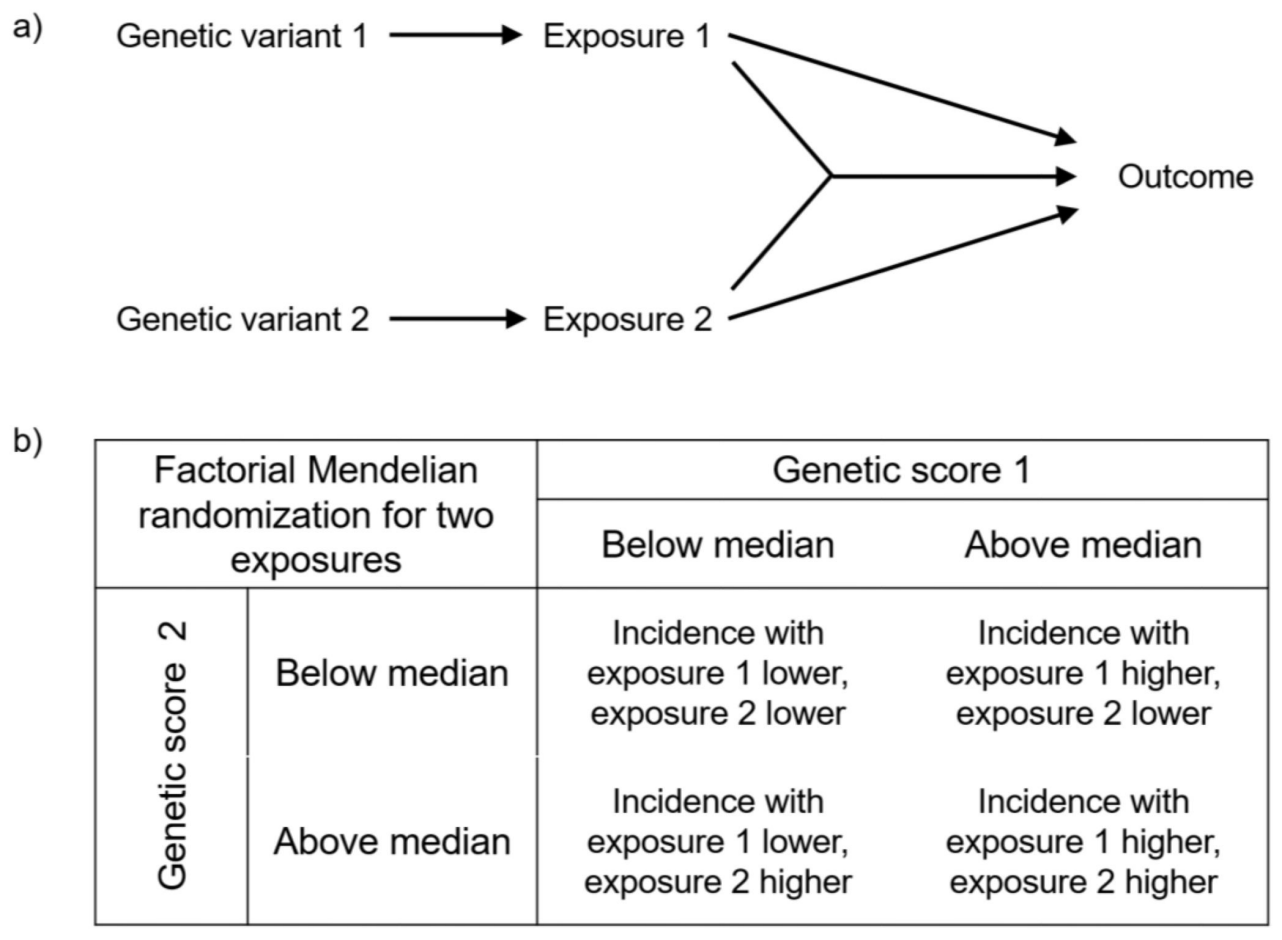
Schematic diagram illustrating factorial Mendelian randomization to assess interactions between exposures: a) diagram indicating causal effects of exposures plus their interaction; b) illustration of the approach as a 2-by-2 design, using dichotomized genetic scores that act as proxies for interventions on exposures 1 and 2.

Another potential complexity in the effect of an intervention is if there is an interaction between two treatments. If separate genetic variants (or sets of variants) are available that proxy distinct interventions, then the statistical interaction between these variants in their association with the outcome can be explored in an approach known as factorial Mendelian randomization, named in analogy with a factorial trial (90) (Figure 5). Initial implementations of factorial Mendelian randomization considered a 2-by-2 design, in which the genetic instruments were dichotomized to divide participants into one of four groups, corresponding to no intervention, intervention A only, intervention B only, and interventions A and B (91). However, while this is an intuitive way of conceptualizing and communicating the analysis, power to detect an interaction is greater when considering the presence of a statistical interaction between the genetic instruments considered as continuous variables (90).

A recent analysis considered interactions between genetic variants in the *IL6R* gene region, representing proxies for intervention on interleukin-6 receptor signalling, and genetic variants in gene regions corresponding to lipid lowering therapies (*PCSK9*, *HMGCR*, *NPC1L1*). No significant interactions were observed in associations with cardiovascular disease risk, suggesting that the effects of interleukin-6 receptor inhibition and lipid lowering therapies on cardiovascular disease are independent with no detectable departure from additivity (92). Such analyses can help understanding the therapeutic potential of novel targets to provide additional benefit in conjunction with existing medicines, although power to detect an interaction is typically low in practice.

## 5 Uncovering tissue specificity

The availability of genetic association data related to gene expression in particular tissues has created the opportunity for Mendelian randomization analyses to be used to reveal the particular body sites or cell types at which proteins may be exerting their relevant biological effects. For example, recent work partitioned BMI-associated genetic variants into two groups based on whether their effects were likely being exerted in the brain or adipose tissue. Specifically, the approach taken was to perform pairwise colocalization analyses at each of the relevant gene regions, considering the traits of BMI and tissue-specific gene expression (93). Consequently, multivariable Mendelian randomization analyses considered the partitioned sets of variants for brain-related BMI variation and adipose-related BMI variation. These analyses provided evidence that effects of BMI reduction on cardiovascular outcomes were more likely to be a consequence of brain-related mechanisms, as compared to those related to effects in the adipose tissue. For drug development, this provides the important insight that perturbing mechanisms affecting appetite and behaviour may be more effective for achieving weight loss than those affecting peripheral adipose tissue metabolism.

In another example, tissue-specific gene expression data have been used to support a role for brain *ACE* expression in the pathophysiology of Alzheimer’s disease (94). Particularly when pursuing drug development, insight into the relevant site of action is paramount for ensuring that any ensuing asset has appropriate pharmacokinetic properties, while concurrently minimizing risk of potential adverse effects in other tissues. However, researchers should be aware that GWAS signals often colocalize with gene expression across multiple tissues – it may be that expression data in sufficient sample sizes for the most relevant tissue or cell type are not available.

A potential limitation of employing variants related to gene expression is that such data are typically taken from donors (often either healthy or deceased) and may not reflect associations observed in disease states. Further, gene expression patterns can be sensitive to environmental factors (such as glycaemic status or pH balance), and thus may not be reflective of the relevant pathophysiological processes (95). A further limitation is that measurements of gene expression in many current datasets are derived from bulk tissues, which are mixtures of different cell types, whereas the relevant effects on expression (and therapy) may be specific to a single or small number of cell types; although single-cell expression datasets are now becoming available (96).

## 6 Investigating heterogeneity in responses

Mendelian randomization investigations typically provide an estimate that represents the population-averaged impact of a shift in the distribution of an exposure (97). However, it may be that the effect of the exposure on the outcome varies between subgroups of the population. By identifying such subgroups, treatments can be targeted towards those who will benefit most.

One scenario that would lead to such heterogeneity is if the effect of the exposure is non-linear. Non-linearity in causal relationships can be investigated in a Mendelian randomization framework, but requires the availability of individual-level data on the genetic variants, exposure, and outcome in a single sample. A popular method for non-linear Mendelian randomization first stratifies the sample based on levels of the exposure, and then conducts separate Mendelian randomization analyses within each stratum (97, 98). An important methodological point is that stratifying on the exposure directly would break randomization and lead to biased estimates in the strata (99). This is because the distribution of the genetic variants would no longer be the same within each stratum, as genetic variants predisposing individuals to higher levels of the exposure would be more common in strata with high levels of the exposure and less common in strata with low levels of the exposure. This is an example of collider bias (100). For this reason, the method first regresses the exposure on the genetic variants, and then stratifies on residual values of the exposure from this regression, as this “residual exposure” is independent of the genetic variants, and hence randomization still holds within strata of the residual exposure. An alternative method, known as PolyMR, also calculates this residual exposure, but then performs a parametric instrumental variable analysis using a polynomial function of the residual exposure to estimate the shape of the non-linear relationship (101).

The residual method was used to investigate the effect of alcohol on cardiovascular disease outcomes (102). A non-linear Mendelian randomization investigation found evidence that the effect of alcohol consumption on coronary artery disease is much stronger at high levels of alcohol consumption compared with at low levels of alcohol consumption, although a harmful effect of alcohol consumption was evident across the whole distribution. This suggests that reductions in alcohol consumption will have the strongest effect on coronary artery disease risk for those with high levels of consumption. In contrast, non-linear Mendelian randomization analyses for the effect of blood pressure on coronary heart disease risk (103), and the effect of average blood glucose levels on coronary heart disease risk have indicated linear causal relationships (104), suggesting that appropriate interventions on these risk factors may be similarly beneficial for the whole population.

Another possibility is that the causal effect of an exposure differs within strata of a measured covariate. Again, stratifying directly on a covariate directly could induce collider bias (105). A similar approach to calculate and then stratify on a residual version of the covariate has been proposed, to stratify on a covariate without inducing collider bias (106). This stratification approach has been used to show that the effect of smoking on risk of bladder cancer is greater for those with low bodyweight (106), and that the effect of interleukin-6 receptor inhibition on coronary heart disease risk is greater for those with high levels of C-reactive protein (107).

An important assumption made by these methods is that the genetic effect on the exposure is linear and constant for all individuals in the population. In many cases, this assumption will be questionable. In other cases, it logically cannot hold; for example, if the exposure has a natural zero level (such as non-consumers for alcohol as an exposure) or is rounded to the nearest whole number. An alternative method has been proposed that stratifies the population without making such strict parametric assumptions (108). This method is implemented by ranking the population twice: first, based on the value of the instrument, and second, based on the value of the exposure. As with the residual method, the strata formed are independent of the genetic variants.

Another possibility is that the effect of an exposure varies within genetic subgroups of the population, an area of research known as “pharmacogenetics”. Genome-wide association studies of response to drug treatment, such as estimated from a randomized clinical trial, have revealed specific variants that associate with treatment response (109). These can also be combined to provide a polygenic treatment response score (110). This could be used to identify potential non-responders to treatment in whom alternative therapies may be preferred (111), or alternatively those who would benefit most from early intervention. This would also be relevant when a treatment has non-negligible side-effects, to compare causal estimates in different subgroups of the population and hence judge whether benefits outweigh risks in each subgroup.

This approach can also be mirrored for cross-sectional observational data in a Mendelian randomization framework using genetic variants as proxies for drug treatments, and investigating gene—gene interactions between the treatment-mimicking instrument and other variants that represent potential effect modifiers. Even if individual variants with strong gene—gene interactions cannot be detected, it is possible to combine signals across sub-GWAS significant variants using a random forest approach to construct a polygenic response score (112). This idea is analogous to the creation of a polygenic risk score, which can provide superior performance for risk prediction compared with approaches that only include GWAS significant variants (113).

## 7 Ancestry-specific considerations

Ancestry has a special place in human genetic investigations, due to the complex interplay of genetics with ethnic identity and cultural practice (114). Typically, Mendelian randomization analyses are conducted in populations from a single ancestry group, under the assumption that this population does not contain substructure (that is, it is a “well-mixed population” (115)). Population stratification can lead to associations between genetic variants and traits that reflect social relationships rather than biological mechanisms. For example, if a genetic variant is more prevalent in a subpopulation that has higher risk of a disease, then the variant will be associated with disease risk. For this reason, it is recommended to adjust for genomic principal components when estimating genetic associations (116). Empirical investigations have provided supportive evidence that population stratification in curated datasets does not lead to substantially more significant associations than would be expected due to chance alone (9). However, investigations in UK Biobank have demonstrated that the assumption of a well-mixed population is violated for this dataset (117). Investigators should be aware of the possibility of genetic associations being biased by population stratification in general, even for datasets that are not large enough for this assumption to be assessed. A further possibility is to conduct Mendelian randomization analyses within families (118); this is particularly important for exposures that are socially patterned compared with those that are biologically determined (119).

Although most Mendelian randomization investigations have been performed in European descent populations, important investigations have been performed in other population groups. For lipoprotein-associated phospholipase A2 (Lp-PLA_2_), the existence of a common inactivating variant in the *PLA2G7* gene region in South Asians enabled investigations to consider a far greater magnitude of change in Lp-PLA_2_ concentrations than would have been possible based on analyses in European descent populations (120). Similarly, the common “alcohol flushing response” gene in the *ALDH2* gene region enables powerful analyses to investigate the effect of alcohol consumption in East Asians (121). There are no genetic variants in Europeans that explain a similar proportion of variance in the distribution of alcohol consumption.

Effect heterogeneity when comparing estimates between population groups can provide mechanistic insights, as well as providing information on the potential benefit of intervention on the exposure in each population. Mendelian randomization analyses of metabolic traits on stroke risk in African ancestry individuals mirrored results in European ancestry individuals (122). However, an analysis considering the effect of lipid traits revealed evidence for a harmful effect of LDL-cholesterol on Type 2 diabetes risk in African ancestry individuals, in contrast to the protective effect in European ancestry individuals (123). This may reflect a stronger representation of lipoprotein lipase-related mechanisms rather than LDL receptor-related mechanisms in African ancestry individuals (124). As a further example, Mendelian randomization estimates for the effect of lipoprotein(a) on coronary heart disease risk were stronger for European ancestry individuals than for African ancestry individuals (125).

A limitation of such analyses is lack of suitable ancestry-specific data for non-European ancestry populations. Allele frequencies and patterns of linkage disequilibrium may differ between populations, with implications for Mendelian randomization and colocalization methods. For example, a genetic variant may have a pleiotropic association in one ancestry group (due to linkage disequilibrium with another functional variant) but not in another group. Low sample size has a dual effect on power in Mendelian randomization investigations: first, genetic associations with the outcome are less precise; and secondly, identified genetic variants typically explain less variability in the exposure. Researchers often face a choice between choosing genetic variants based on larger European ancestry datasets, or based on smaller ancestry-specific datasets. The former option may lead to a greater number of selected variants, but there is no guarantee that genetic predictors of the exposure selected in Europeans will be the optimal predictors of the exposure in another ancestry group (126). However, efforts are underway to gather such data, as well as to develop suitable tools for the analysis of non-European data (127).

## 8 Predicting the magnitude of effect

Mendelian randomization typically compares genetically-defined subgroups of the population that have different trajectories in their average level of the exposure since childhood. Hence, estimates typically reflect the impact of life-long differences in an exposure. However, it may be that the impact of sustained differences in an exposure differs from the impact of short-term interventions achievable in a clinical trial (128, 129).

When designing a clinical trial for lipoprotein(a) lowering, an important question is the trial inclusion criterion relating to lipoprotein(a) levels (130). The distribution of lipoprotein(a) in European ancestry individuals is highly skewed, with median levels at around 30 mg/dL, but as much as 1000-fold differences between individuals (131). Recruiting individuals at the median lipoprotein(a) value would limit the maximum possible benefit of lipoprotein(a) lowering therapies, as the maximum absolute reduction in lipoprotein(a) levels would be 30 mg/dL. In contrast, the maximum absolute reduction in lipoprotein(a) levels would be over three times greater for an individual with lipoprotein(a) levels of 100 mg/dL. Predicting the magnitude of effect of lipoprotein(a) lowering was critically important to the design of the trial in order to focus recruitment on individuals having a potential detectable benefit of lipoprotein(a) lowering, and hence maximize power, given that any trial is finite in sample size.

Lipoprotein(a) is particularly amenable to Mendelian randomization investigations, as it is highly heritable (132, 133). Genetic variants having a wide range of magnitudes of association with lipoprotein(a) levels were able to demonstrate a log-linear relationship between the genetic association with lipoprotein(a) levels and the genetic association with coronary heart disease risk, suggesting that the potential benefit of intervention was proportional to the absolute change in lipoprotein(a) levels (76).

To estimate the potential benefit of lipoprotein(a) lowering in a short-term trial, investigators compared Mendelian randomization estimates for LDL-cholesterol lowering to trial estimates for LDL-cholesterol lowering (76). The same ratio of life-long to short term estimates observed for LDL-cholesterol lowering was assumed to hold for lipoprotein(a) lowering. This may be reasonable given their similarities as circulating lipid traits that are believed to causally affect cardiovascular risk. This enabled investigators to estimate the potential benefit of lipoprotein(a) lowering in a short-term trial, by multiplying the Mendelian randomization estimate for lipoprotein(a) by this ratio. Consistent with these calculations, the HORIZON trial used a cut-off of 70 mg/dL for participant inclusion; only individuals with lipoprotein(a) levels above this threshold were invited to participate in the trial (134).

A limitation of this investigation is that it only considered lipoprotein(a) concentrations and not apolipoprotein(a) isoform size; a previous Mendelian randomization analysis indicated that apolipoprotein(a) isoform size is an independent causal risk factor for coronary artery disease (135). This is a potential reason why Mendelian randomization estimates for lipoprotein(a) concentration differ between European and African ancestry populations (125), as the distributions of apolipoprotein(a) isoform size and the number of kringle IV repeats differ between the ancestry groups (136).

## 9 Understanding timings of interventions

Mendelian randomization analyses can investigate the impact of interventions on an exposure during critical time-periods. A relatively under-studied population in the context of drug development efforts is women of childbearing age and pregnant women. Due to risks to the fetus, clinical study of drug effects in this population is more challenging, with the result that fewer effective therapies are available for pregnant women. To combat this, genetic data may be used to inform on the relative safety of interventions. For example, genetic evidence has suggested that blood pressure lowering may reduce risk of pre-eclampsia or eclampsia in pregnant women (137), a finding that was subsequently supported in evidence from clinical trials (138). More recently, genetic data have been used to investigate the comparative safety of beta-blocker and calcium channel blocker antihypertensive drugs in pregnancy, with the evidence suggesting that beta-blocker effects may lower offspring birthweight (139).

An extension of this notion is to consider how interventions in the exposure at different time-points may affect the outcome. By understanding the relationship between time and causal effects, we can better consider how the timing of an intervention will impact clinical outcomes.

If genetic variants are available that are more strongly associated with the exposure at specific time-points than others, then the values of the exposure at different time-points can be used as separate risk factors in a multivariable Mendelian randomization analysis. For example, investigators have considered BMI measured during early life and during later life as separate risk factors, and assessed whether genetically-predicted values of early life and later life BMI were associated with coronary artery disease risk (140). A positive univariable association between genetically-predicted early life BMI and coronary artery disease risk was interpreted as evidence that early life BMI is a causal risk factor for coronary artery disease. Lack of independent association between genetically-predicted early life BMI and coronary artery disease risk in a multivariable analysis that adjusted for genetically-predicted later life BMI provided evidence that early life BMI does not have a direct effect on coronary artery disease risk. The effect of early life BMI appears to be mediated via later life BMI. This has translational relevance, as it implies that for a general population, bodyweight should be pharmacologically lowered in adult life for the purposes of reducing cardiovascular risk, and not in early life.

As a note of caution, such analyses are likely to suffer from model misspecification, as it is unlikely that disease risk is a discrete function of the exposure at the fixed timepoints considered in the multivariable model (141). We would therefore recommend that these analyses are only conducted when values of the exposure at different timepoints can reasonably be interpreted as distinct risk factors, and not simply measurements of the same risk factor at different timepoints. Empirical investigations have shown that results in the latter case can be misleading, with no guarantee that estimates from multivariable Mendelian randomization correspond to the true pattern of the time-varying effect of the exposure (141).

A further potential area of investigation is effects on disease progression or disease survival. While Mendelian randomization analyses have suggested that BMI is a protective risk factor for breast cancer (142, 143), separate analyses have indicated that BMI is a harmful risk factor for breast cancer progression (144). Mendelian randomization analyses for disease progression are difficult to implement, as there is a natural selection event that may induce bias: disease progression can only be measured in individuals who have had an initial disease event (145, 146). If the exposure influences the risk of the disease, then this can lead to collider bias (45). Additionally, disease progression cohorts are typically older and less healthy, which can lead to substantial survival bias. Few GWAS of disease progression are available (147). However, the increasing availability of large biobank cohorts of individuals who are (relatively) disease-free at baseline and have extensive follow-up data make this a potential fertile ground for future investigations (148).

### Limitations and future directions

The limitations of Mendelian randomization have been widely discussed since the conception of the approach (149, 150). We focus here on limitations of Mendelian randomization for testing a causal hypothesis, and those particularly relevant to drug discovery and development; several other limitations have already been discussed in the relevant sections. As per Section 8, there are many additional reasons why Mendelian randomization is not well-suited for estimation of the magnitude of a causal effect (principally, it considers life-long variability in traits due to genetic variation, whereas trials assess the impact of short-term changes (128, 129)).

Causality cannot be demonstrated directly from observational data. Any causal claim must rely on an untestable assumption. In the case of Mendelian randomization, we assume that genetic variants used in the analysis satisfy the assumptions of an instrumental variable: they are associated with the exposure, not associated with the outcome via a confounding pathway, and can only influence the outcome via their effect on the exposure. As an alternative phrasing of these assumptions, we assume the genetic variants are distributed “as if randomly” in the population (that is, independently of competing risk factors) and gene—environment equivalence (that is, the result of inheriting a genetic variant is qualitatively equivalent to the proposed intervention that we are assessing). If any of these assumptions are violated, Mendelian randomization investigations can be misleading.

For some exposures and corresponding interventions, we have plausible genetic instruments. For others, either we do not, or else there is uncertainty in the extent to which the genetic variants mimic the intervention. For example, glycated haemoglobin and urinary sodium excretion can be considered as sentinel traits (positive control effects) for sodium-glucose co-transporter-2 (SGLT2) inhibitors; that is, any variant that mimics SGLT2 inhibitors should be associated with both glycated haemoglobin and urinary sodium levels. A recent investigation of variants in the *SLC5A2* gene region did not find any common variants that were associated with both glycated haemoglobin and urinary sodium excretion, and so could be used as instruments in Mendelian randomization analyses (151). As a further example, the precise mechanism by which metformin exerts effects on glycaemic control is unclear. While a recent investigation considered genetic variants relating to levels of postulated downstream protein targets of metformin (152), the direct relevance of this analysis to the impact of metformin in clinical practice is uncertain. In other cases, it may be unclear what aspect of intervention on the exposure is proxied by the genetic variants. For example, HDL particles are heterogeneous in their protein and enzymatic content. The extent to which this function is captured by genetic predictors of HDL-cholesterol concentrations is unclear. Some drug targets, such as the target of calcium channel blockers, are made up of more than one protein. Again, the extent to which the totality of the drug effect is mimicked by genetic variants in gene regions relating to individual protein targets is questionable. Further still, many neuropsychiatric drugs have wide-ranging effects on a variety of targets.

In isolation, Mendelian randomization investigations into drug targets may be of limited value for unravelling the optimal mode of action for a pharmacological intervention. Specifically, if an instrument for drug target perturbation relates to risk of a particular disease, further functional insights will be necessary to uncover the mechanism by which this effect may be occurring. While additional Mendelian randomization analyses, including exploration of potential mediating pathways, may be informative in this respect, such genetic analyses must typically still be supplemented with experimental approaches that offer complementary insights.

We note that a positive finding from a well-conducted Mendelian randomization analysis is no guarantee that intervention on the corresponding pathway will yield a successful drug. For example, interventions on the pathway may have undesired adverse effects, or may not result in sufficient clinical benefit for the target disease. A relevant example is CETP inhibition; although variants in the *CETP* gene region are associated with lower coronary heart disease risk (153), and trials of anacetrapib demonstrated reduced incidence of major coronary events (154), these findings have so far been insufficient for the drug to be employed in routine clinical practice.

In conclusion, the availability of large-scale genetic data has created unprecedented opportunity to offer insight for drug target identification and consequent clinical development. In order to maximize the potential of these data, it is equally important that appropriate methods be used to capitalize on these learnings. There are many ways that genetic variants can be used to provide evidence not only on the causal nature of a target, but also focused evidence that addresses questions of translational relevance and guides the drug development process. With the advent of publicly-available data from GWAS studies as well as large population-based cohort studies with concomitant phenotypic and genetic data (such as UK Biobank (155)), the limiting factor for such investigations is often not data availability, but analyst skill and time. As the techniques discussed in this review become more familiar to investigators, we hope to see their application becoming routine in all stages of the drug development pipeline.

## Figures and Tables

**Table 1:** Some published examples where human genetic data have been leveraged to facilitate and inform drug development in cardiometabolic disease.

Target	Gene	Outcome	Conclusions from genetic evaluation	Clinical validation of genetic evidence
**Coagulation factor X**	*F10*	Cardiovascular and thrombotic disease	Repurposing potential of factor Xa inhibitors in other cardiovascular disease subtypes (21)	Phase III clinical trial (22)
**Coagulation factor XI**	*F11*	Cardioembolic stroke	Protective effects of factor XI inhibition in cardioembolic stroke (36)	Phase III trial evidence in venous thromboembolism (163)
**Fibroblast growth factor 21 (FGF21)**	*FGF21*	Non-alcoholic steatohepatitis	Favourable effects of circulating FGF21 on cardiometabolic biomarkers (164)	Phase II clinical trial (165)
**Glucagon-like peptide 1 (GLP1)**	*GLP1R*	Heart failure	Protective effects of GLP1 agonism in heart failure (166)	Phase III trial underway (NCT01800968)
**Glucose-dependent insulinotropic polypeptide (GIP)**	*GIP*	Cardiometabolic disease	Favourable effects of GIP agonism on bodyweight, lipid traits, coronary artery disease and inflammation (167)	Phase III clinical trials (168, 169)
**Interleukin 6 receptor (IL6R)**	*IL6R*	Cardiovascular disease, severe covid-19, adverse effects, repurposing potential and biomarkers	Beneficial effects of IL6R inhibition in severe COVID-19 (72), increased risk of infectious, allergic and autoimmune disease (170), identify biomarkers to measure efficacy (170), repurposing potential in atherosclerotic disease (171)	Phase II and III clinical trial evidence (74, 172)
**Proprotein convertase subtilisin/kexin type 9 (PCSK9)**	*PCSK9*	Cardiovascular disease	Protective effects of PCSK9 inhibition (173)	Phase III clinical evidence (174)
**Niemann-Pick C1-like 1 (NPC1L1)**	*NPC1L1*	Coronary heart disease	Protective effects of NPC1L1 inhibition (175)	Phase III clinical evidence (176)
**Cholesteryl ester transfer protein (CETP)**	*CETP*	Cardiovascular disease	Protective effects of CETP inhibition on cardiovascular disease (177)	Phase III clinical evidence (154)

**Table 2:** List of software packages for Mendelian randomization (MR) and related methods discussed in this review. This is not a comprehensive list of all MR methods, but covers the methods most commonly used in the literature.

Approach	Package name	Weblink
Multiple methods	MendelianRandomization	https://cran.r-project.org/web/packages/MendelianRandomization/
Multiple methods	TwoSampleMR	https://github.com/MRCIEU/TwoSampleMR
Outlier-robust estimation	MR-PRESSO	https://github.com/rondolab/MR-PRESSO
Colocalization	coloc	https://github.com/chr1swallace/coloc
Rare variant burden testing	SKAT	https://cran.r-project.org/web/packages/SKAT/
Rare variant burden testing	regenie	https://rgcgithub.github.io/regenie/
Robust estimation for multivariable MR	Robust MVMR	https://github.com/aj-grant/robust-mvmr
Variable selection	MR-BMA	https://github.com/verena-zuber/demo_AMD
Factor-based *cis*-MR	con-cis-MR	https://github.com/ash-res/con-cis-MR
Network *cis*-MR	TwoStepCisMR	https://github.com/bar-woolf/TwoStepCisMR/wiki
Clustering variants based on outcome associations Clustering variants	MRClust	https://github.com/cnfoley/mrclust
based on trait associations	NAvMix	https://github.com/aj-grant/navmix
Non-linear residual method (individual-level data)	nlmr	https://github.com/jrs95/nlmr
Non-linear residual and doubly-ranked methods	SUMnlmr	https://github.com/amymariemason/SUMnlmr
Non-linear polynomial method	PolyMR	https://github.com/JonSulc/PolyMR

## Data Availability

Most methods listed in this review can be implemented using summarized genetic associations (156), representing the beta-coefficients and standard errors from univariable regression on each genetic variant in turn, and which have been made publicly available by many large GWAS consortia (157) and for large population-based biobanks (158–160). These methods can be implemented using the MendelianRandomization (161) and TwoSampleMR (16) packages for R. An exception is non-linear Mendelian randomization, which requires individual participant data (162). A full list of software packages and links is provided in Table 2.
